# Preventing sudden unexpected postnatal collapse in term and late preterm newborn infants: a surveillance protocol

**DOI:** 10.1186/1824-7288-40-S2-A5

**Published:** 2014-10-09

**Authors:** Riccardo Davanzo, Laura Travan, Giuseppina Verardi, Elisa Corubolo, Angela De Cunto, Giulia Paviotti, Tamara Strajn, Francesca Marrazzo, Pierpeolo Brovedani, Jaquelyn Kennedy, Enrica Causin, Sergio Demarini

**Affiliations:** 1 Perinatal Medicine, Institute for Maternal and Child Health IRCCS “Burlo Garofolo”, Trieste, Italy

## 

Early and prolonged skin-to-skin contact (SSC) after birth between a mother and her newborn has been shown to generate beneficial effects on mother-infant relationship and breastfeeding. SSC may ease the infant’s transition to extra uterine life and helps to regulate the infant’s body temperature and nursing behavior.

However, reports of sudden unexpected postnatal collapse (SUPC) soon after birth, in healthy term and late preterm neonates, in association with skin-to-skin contact, have raised concerns about the safety of this practice.

Based on the available evidence, the working group on breastfeeding of the Maternal and Child Health Institute of Trieste (Italy) developed a surveillance protocol to be implemented in the Delivery Room and Postnatal Ward. The aim of our protocol is: 1) promoting safe mother- infant bonding 2) establishing successful early breastfeeding and 3) correcting the risk factors for sudden unexpected postnatal collapse (SUPC). This protocol is especially focused on the first 2 hours of life, when about 1/3 of SUPC occur, but extends to the whole duration of the infant stay in the maternity ward.

The following interventions will be undertaken: 1. antenatal and early postnatal oral and written information to parents about: a) the risk of bed-sharing b) avoidance potentially suffocating infant positions (i.e. mouth/nose obstruction) c) the need of an adequate supervision of the infant in the first hours/days after birth 2. periodical assessment (position, colour, breathing) of the infant (at 10, 30, 60, 90 and 120 minutes of life) by midwives in the delivery room 3. discouragement of bed-sharing 4. encouragement of skin-to-skin contact only when mothers are fully awake 5. avoidance of mothers left alone with the baby in the first hours after birth particularly during skin-to-skin contact and first breastfeeding attempts.

As there is no evidence of effective interventions to prevent SUPC, our protocol has been written as a potential best practice. Evidence of its clinical effectiveness is obviously needed.

